# Enhancing anticancer activity of macrophages through rational drug combinations

**DOI:** 10.1172/JCI180512

**Published:** 2024-05-01

**Authors:** Gordon B. Mills, Marilyne Labrie

**Affiliations:** 1 Knight Cancer Institute, Oregon Health & Science University, Portland, Oregon, USA.; 2 Department of Immunology and Cell Biology, Faculty of Medicine and Health Sciences, Université de Sherbrooke, Sherbrooke, Quebec, Canada.; 3 Centre de Recherche du Centre Hospitalier de l’Université de Sherbrooke (CRCHUS), Sherbrooke, Quebec, Canada.; 4 Institut de Recherche sur le Cancer de l’Université de Sherbrooke (IRCUS), Sherbrooke, Quebec, Canada.

## Abstract

Targeting tumor-associated macrophages (TAMs) is an emerging approach being tested in multiple clinical trials. TAMs, depending on their differentiation state, can exhibit pro- or antitumorigenic functions. For example, the M2-like phenotype represents a protumoral state that can stimulate tumor growth, angiogenesis, metastasis, therapy resistance, and immune evasion by expressing immune checkpoint proteins. In this issue of the *JCI*, Vaccaro and colleagues utilized an innovative drug screen approach to demonstrate that targeting driver oncogenic signaling pathways concurrently with anti-CD47 sensitizes tumor cells, causing them to undergo macrophage-induced phagocytosis. The combination treatment altered expression of molecules on the tumor cells that typically limit phagocytosis. It also reprogrammed macrophages to an M1-like antitumor state. Moreover, the approach was generalizable to tumor cells with different oncogenic pathways, opening the door to precision oncology–based rationale combination therapies that have the potential to improve outcomes for patients with oncogene-driven lung cancers and likely other cancer types.

## Macrophage checkpoint inhibitors expand the immunotherapy arsenal

T cell checkpoint inhibitors (ICIs) have transformed the therapy landscape for a number of diseases; however, only a fraction of patients with cancer receive substantial benefit from ICIs. While effective biomarkers for ICI activity remain elusive, their activity hinges on several factors, including the tumor mutation burden and the expression of tumor neoantigens, the expression of immune checkpoint proteins, intratumoral heterogeneity, and the overall immune landscape (1, 2). Regrettably, these factors limit the activity of current ICIs targeting T cell function in many cancers. For example, a subset of patients with non–small cell lung cancer (NSCLC) who have oncogenic mutations in genes, such as EGFR, ALK, or KRAS, typically respond poorly to ICIs (3).

Tumor-associated macrophages (TAMs) have emerged as critical mediators of tumor initiation, progression, and therapeutic resistance (1, 4). Macrophages exhibit remarkable phenotypic plasticity that allows them to switch among distinct functional states in response to cytokines, tumor microenvironment cues, and cell interactions (1, 4). They were initially classified into M1 and M2 subtypes based on in vitro studies of murine macrophages, with M1 macrophages described as antiinflammatory and M2 macrophages defined as proinflammatory or protumorigenic. However, in vivo and human studies suggest that the original classification of M1 and M2 was simplistic, and that macrophage subtypes express different functional characteristics. The terms M1-like and M2-like have been adopted by the community to reflect functional characteristics rather than specific markers or macrophage subtypes (5). More recent large-scale microenvironment transcriptional profiling studies have suggested that there are multiple different macrophage subtypes and that they most commonly express characteristics that are not solely reflected by the M1-like and M2-like designations (6, 7).

TAMs can enhance neoantigen presentation through cancer cell phagocytosis, potentially boosting adaptive T cell immunity (1, 4). This function is most strongly associated with a M1-like phenotype. Thus, pharmacologically increasing phagocytic capacity and antigen presentation to T cells has been proposed as an attractive therapeutic approach, especially in tumors with low tumor mutation burden. In the tumor microenvironment, cancer cells can inhibit macrophage phagocytic activity through the expression of “don’t-eat-me” checkpoint proteins (i.e., CD47 and CD24). Anti-CD47 antibodies that block the interaction of CD47 with its ligand SIRPα, expressed on macrophages, have shown promising activity in preclinical cancer models by triggering cancer cell phagocytosis and enhancing adaptive immune responses against the cancer cells. Unfortunately, clinical trial results have so far been suboptimal owing to limited drug response duration and accrued toxicity (8). Anti-CD24 antibodies that block the interaction with its ligand Siglec-10, expressed on macrophages and other innate immune cells, increase phagocytosis of cancer cells by macrophages, with encouraging results in early-phase clinical trials (9, 10). Indeed, ClinicalTrials.gov lists more than 20 trials as completed or underway that explore the clinical utility of targeting CD47 and CD24, with most of these being monotherapy.

**Figure 1:**
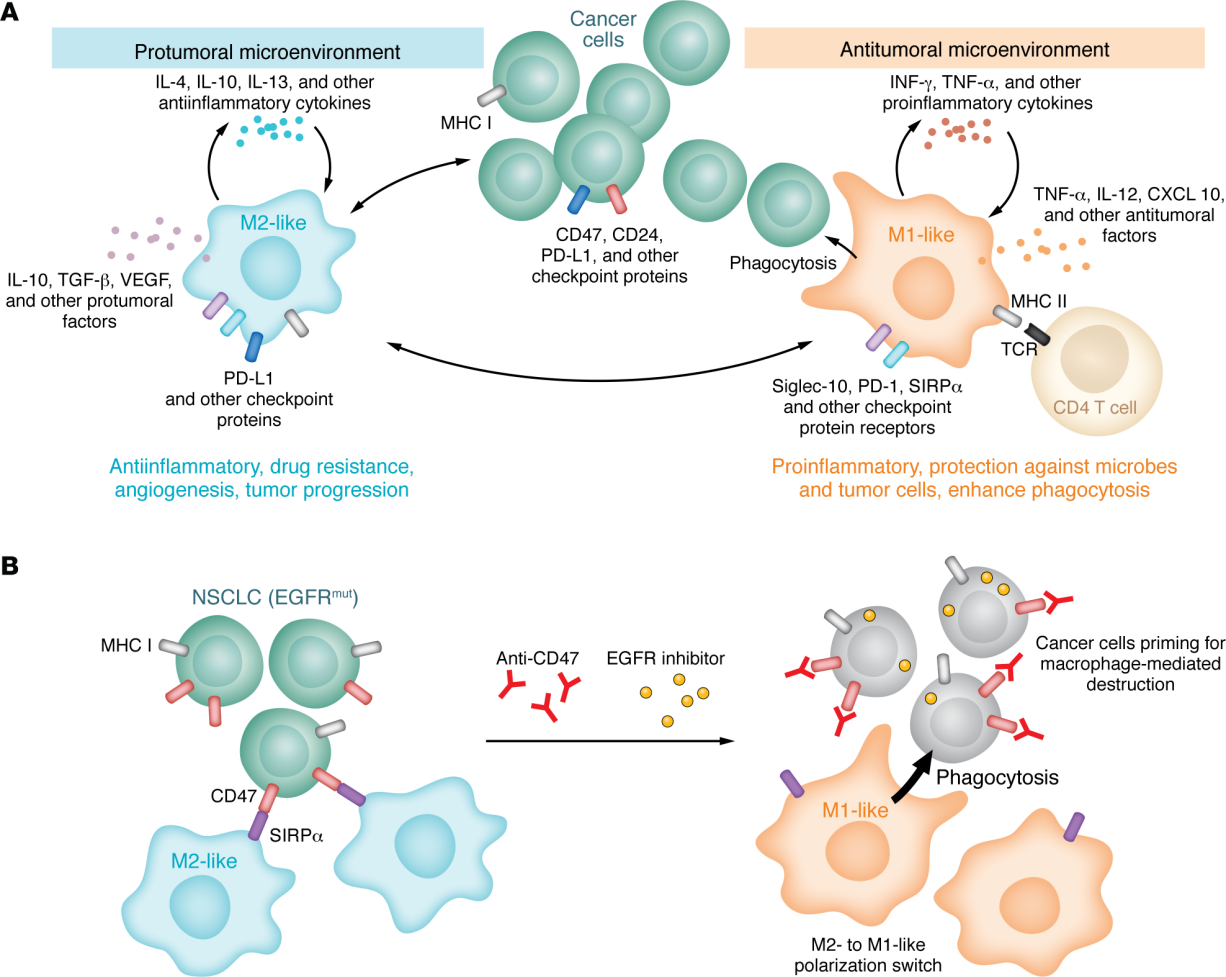
TAM polarization and function affect anticancer activity. (**A**) Cancer cells can modulate macrophage function by expressing macrophage checkpoint proteins and through the remodeling of the tumor microenvironment. The presence of antiinflammatory cytokines polarizes TAMs, causing them to adopt an M2-like phenotype. These macrophages secrete proangiogenic and antiinflammatory factors, stimulating tumor progression. Conversely, in the presence of proinflammatory cytokines, TAMs harbor an M1-like phenotype and secrete proinflammatory factors that exert an antitumoral function. (**B**) EGFR-driven NSCLC cells express high levels of CD47 and MHC I. Targeting the cancer cells with EGFR inhibitors and anti-CD47 primes them for macrophage-mediated destruction and reverts the M2-like phenotype of macrophages to a M1-like phenotype. As a result, cancer cells are subject to phagocytosis, which eliminates persister cells.

The tumor ecosystem, with bidirectional interactions between tumor cells and macrophages as well as the effects of cytokines, can influence macrophage polarization into different subtypes (Figure 1A). For example, an IFN-γ– and TNF-α–rich tumor microenvironment increases polarization of macrophages toward a proinflammatory M1-like phenotype. Conversely, in tumors where cancer cells secrete antiinflammatory cytokines such as IL-4, IL-10, or IL-13, macrophages are pushed toward a protumoral M2-like phenotype (1). Interestingly, although still poorly characterized, recent data have demonstrated that small-molecule drugs used to target oncogene-driven cancer cells can also trigger macrophage phenotypic remodeling through direct or indirect effects. Drugs can also modulate the microenvironment to reduce or increase recruitment of different macrophage subtypes to the tumor site. For example, the BCL-2 inhibitor APG-2575 has direct antitumor activity in hematologic malignancies (11), but it also alters macrophage polarization, pushing them toward a M1-like phenotype (12). Other drugs, such as the PI3Kγ inhibitor IPI-549, reduce proliferation of cancer cells by reshaping the tumor microenvironment and pushing macrophages to polarize into a M1-like phenotype (13). In this case, the inhibitor has a direct effect on myeloid cells and increases tumor responses to ICIs (13).

## Exploiting phenotypic plasticity of macrophages

Data supporting anti-CD47 as an active therapy combined with the potential of small-molecule therapies to modify macrophage polarity supported development of a platform that would identify combination therapies that could increase (or decrease) the activity of anti-CD47 in NSCLC. This strategy could have clinical relevance, as TAMs, likely M2 like, have been shown to drive resistance to EGFR inhibitors in NSCLC (14). Vaccaro and colleagues implemented an innovative drug screening platform to find therapies that have the ability to increase cancer cell susceptibility to macrophage-induced destruction (15). A screen of 500 FDA-approved drugs in combination with anti-CD47 was performed in the presence of macrophages differentiated toward a M2-like phenotype. This screen identified small molecules that decreased the activity of anti-CD47 (including steroids, retinoids, and anthracyclines) and, importantly, a class of molecules that increased the activity of anti-CD47 in an EGFR-driven NSCLC model.

## Macrophage-mediated destruction and adaptive immune response

In this issue of the *JCI*, Vaccaro and colleagues first identified EGFR inhibitors that increased the activity of anti-CD47 in an EGFR-driven NSCLC model (Figure 1B). Then, they showed that directly targeting diverse cancer driver mutations, including EGFR, ALK, and RAS, as well as their downstream signaling pathways, could prime cancer cells for destruction by macrophages and remodel macrophage polarization (15). The concept was validated in multiple model systems, importantly, demonstrating potential generalizability. Crucially, the combination of inhibitors that targeted cancer drivers with anti-CD47 eliminated persister cells in vivo and resulted in tumor regressions and cures in a number of relevant in vivo model systems. The authors explored several potential mechanisms; however, the comprehensive molecular mechanism underlying this activity warrants further exploration to facilitate optimal clinical implementation. Interestingly, there was a direct correlation between EGFR activating mutations and elevated levels of CD47 and MHC I molecules on cancer cells. Consequently, blocking CD47 could potentially increase the ability of CD8^+^ T cells to detect cancer neoantigens presented by the MHC I complex, thereby promoting an adaptive immune response against the cancer cells (16). This concept is further supported by a study conducted on skin cancer, which demonstrated that EGFR inhibitors modulate MHC I protein levels (17), thereby favoring antitumor immune responses. Vaccaro and colleagues also provided evidence that the combination therapy could shift the polarization of macrophages from an M2-like protumorigenic to an M1-like antitumorigenic phenotype (15). This transformation appears to be directly induced by the macrophages themselves, as they were observed to secrete proinflammatory cytokines in the presence of the combination therapy, even in the absence of cancer cells. Together, the generalizability, as well as the remarkable in vivo efficacy, warrants exploration in clinical trials with a precision oncology approach evaluating the appropriate combination therapies in oncogene-driven cancers, with an emphasis on both efficacy and toxicity.

## Version 1 05/01/2024

Electronic publication

## References

[1] Zhou B (2024). Targeting the macrophage immunocheckpoint: a novel insight into solid tumor immunotherapy. Cell Commun Signal.

[2] Wang P (2021). Beyond tumor mutation burden: tumor neoantigen burden as a biomarker for immunotherapy and other types of therapy. Front Oncol.

[3] Chen J (2024). Efficacy of immunotherapy in patients with oncogene-driven non-small-cell lung cancer: a systematic review and meta-analysis. Ther Adv Med Oncol.

[4] Pittet MJ (2022). Clinical relevance of tumour-associated macrophages. Nat Rev Clin Oncol.

[5] Monnier M (2022). Antitumor strategies targeting macrophages: the importance of considering the differences in differentiation/polarization processes between human and mouse macrophages. J Immunother Cancer.

[6] Cheng S (2021). A pan-cancer single-cell transcriptional atlas of tumor infiltrating myeloid cells. Cell.

[7] Qian J (2020). A pan-cancer blueprint of the heterogeneous tumor microenvironment revealed by single-cell profiling. Cell Res.

[8] Osorio JC (2023). The antitumor activities of anti-CD47 antibodies require Fc-FcγR interactions. Cancer Cell.

[9] Li X (2024). Targeting CD24/Siglec-10 signal pathway for cancer immunotherapy: recent advances and future directions. Cancer Immunol Immunother.

[10] Barkal AA (2019). CD24 signalling through macrophage Siglec-10 is a target for cancer immunotherapy. Nature.

[11] Deng J (2022). Lisaftoclax (APG-2575) is a novel BCL-2 inhibitor with robust antitumor activity in preclinical models of hematologic malignancy. Clin Cancer Res.

[12] Luo F (2024). The BCL-2 inhibitor APG-2575 resets tumor-associated macrophages toward the M1 phenotype, promoting a favorable response to anti-PD-1 therapy via NLRP3 activation. Cell Mol Immunol.

[13] De Henau O (2016). Overcoming resistance to checkpoint blockade therapy by targeting PI3Kγ in myeloid cells. Nature.

[14] Cheng D (2023). Tumor-associated macrophages mediate resistance of EGFR-TKIs in non-small cell lung cancer: mechanisms and prospects. Front Immunol.

[15] Vaccaro K (2024). Targeted therapies prime oncogene-driven lung cancers for macrophage-mediated destruction. J Clin Invest.

[16] Rock KL (2016). Present yourself! By MHC Class I and MHC Class II molecules. Trends Immunol.

[17] Pollack BP (2011). Epidermal growth factor receptor inhibition augments the expression of MHC class I and II genes. Clin Cancer Res.

